# Spatio-temporal pattern, matching level and prediction of ageing and medical resources in China

**DOI:** 10.1186/s12889-023-15945-9

**Published:** 2023-06-15

**Authors:** Zhenyan Wang, Wei Ye, Xicheng Chen, Yang Li, Ling Zhang, Fang Li, Ning Yao, Chengcheng Gao, Pengyu Wang, Dong Yi, Yazhou Wu

**Affiliations:** 1 Department of Health Statistics, College of Preventive Medicine, Army Medical University, NO.30 Gaotanyan Street, Shapingba District, Chongqing, 400038 China; 2 Department of Health Education, College of Preventive Medicine, Army Medical University, NO.30 Gaotanyan Street, Shapingba District, Chongqing, 400038 China

**Keywords:** Population ageing, Medical resources, Spatio-temporal distribution trends, Interaction effect, Regression prediction

## Abstract

**Objective:**

Population ageing, as a hot issue in global development, increases the burden of medical resources in society. This study aims to assess the current spatiotemporal evolution and interaction between population ageing and medical resources in mainland China; evaluate the matching level of medical resources to population ageing; and forecast future trends of ageing, medical resources, and the indicator of ageing-resources (IAR).

**Methods:**

Data on ageing (EPR) and medical resources (NHI, NBHI, and NHTP) were obtained from China Health Statistics Yearbook and China Statistical Yearbook (2011–2020). We employed spatial autocorrelation to examine the spatial–temporal distribution trends and analyzed the spatio-temporal interaction using a Bayesian spatio-temporal effect model. The IAR, an improved evaluation indicator, was used to measure the matching level of medical resources to population ageing with kernel density analysis for visualization. Finally, an ETS-DNN model was used to forecast the trends in population ageing, medical resources, and their matching level over the next decade.

**Results:**

The study found that China's ageing population and medical resources are growing annually, yet distribution is uneven across districts. There is a spatio-temporal interaction effect between ageing and medical resources, with higher levels of both in Eastern China and lower levels in Western China. The IAR is relatively high in Northwest, North China, and the Yangtze River Delta, but showed a declining trend in North China and the Yangtze River Delta. The hybrid model (ETS-DNN) gained an R^2^ of 0.9719, and the predicted median IAR for 2030 (0.99) across 31 regions was higher than the median IAR for 2020 (0.93).

**Conclusion:**

This study analyzes the relationship between population ageing and medical resources, revealing a spatio-temporal interaction between them. The IAR evaluation indicator highlights the need to address ageing population challenges and cultivate a competent health workforce. The ETS-DNN forecasts indicate higher concentrations of both medical resources and ageing populations in eastern China, emphasizing the need for region-specific ageing security systems and health service industries. The findings provide valuable policy insights for addressing a hyper-aged society in the future.

## Introduction

The population ageing problem has become increasingly severe worldwide [1, 2]. In 2020, China's elderly population ratio (EPR), the proportion of the total population aged 65 and over, is about 13.52%, rising by approximately 4.62% since 2010 [3]. During this decade, China's life expectancy increased from 74.83 years to 77.93 years [4]. The elderly population shows an imbalanced regional and urban–rural distribution: the EPR in the east is significantly higher than in the west, with a regional gradient from east to west; the EPR in the countryside is 17.72%, 6.61% higher than that in the towns [5]. China's ageing population will continue to be on a steady rise. The accelerated growth of the elderly population has led to a remarkable boost in demand for medical resources among the elderly [6].

In recent years China has significantly improved its medical technology capabilities and the quality of healthcare. According to the National Ageing Development Bulletin 2020, there is an increasing trend in the number of health institutions, beds in health institutions, and health technicians in the country [7]. In 2020, the number of health institutions nationwide reached 1.023 million, representing an increase of 1.5% year-over-year (YoY). The number of beds in health institutions aggregated to 9.11 million, exhibiting a YoY increase of 3.4%. However, it is noted that the growth rate of the number of beds in health institutions is showing a tendency towards slowing down. Additionally, the number of health technicians reached 10.66 million, with a YoY increase of approximately 5.0%. However, China's rapid ageing has given the healthcare system insufficient time to adjust and respond. Furthermore, the imbalanced urban–rural and regional distribution of the elderly population has challenged the existing layout of medical resource allocation and raised the demand for additional medical resources [8, 9].

Comprehensive public health measures are necessary to address the global phenomenon of population ageing, as stated in the WHO World Report on Ageing and Health [10]. There are also growing concerns in China regarding equity and efficiency in the allocation of health resources and the utilization of health services. Since 2009, China has actively engaged in medical reform in response to the increasing demand for healthcare services from the elderly population [11]. For example, the "integration of medical treatment and elderly support" reform promotes the embedding of medical and health services into the community and institutional care for the elderly [12]. Recent reforms in public hospitals and the construction of a hierarchical healthcare system have provided a strong impetus for the development of senior healthcare [13]. To a certain extent, these reforms have the potential to mitigate the the healthcare demands of the ageing population, enhance the prioritization of elderly patients, and optimize the utilization of medical resources for the elderly. The implementation of such reforms necessitates a substantial investment in healthcare services and the implementation of a well-planned allocation strategy for medical resources. Healthy China 2030 [14], as a comprehensive and ambitious initiative, was then proposed to improve the health and well-being of the nation's population, with a particular focus on the ageing population. However, there are two prevalent challenges [15, 16] that persist. Firstly, despite the recent reforms, the overall availability of medical resources remains inadequate due to the substantial population size in China. Secondly, there is a marked disparity in the distribution of medical resources across the provinces, resulting in regional imbalances.

China’s medical resource allocation has been found to be inadequate and imbalanced in previous studies [15, 16]. Although the equity of medical resources per capita in China has gradually increased, there is a clear geographical imbalance in their distribution. Presently, China has nurtured a favorable policy environment for the promotion of healthy ageing [17]. In spite of this, it remains unclear whether the medical resource allocation is adequate to meet the demands of an ageing society, thus indicating the need for further research. The exploration of the spatio-temporal matching of population ageing and medical resources not only allows for assessing the inadequacy of medical resources in response to the growing ageing population, but also informs forward-looking policy formulation at the national level [18]. In addition, exploring the spatio-temporal evolution and interaction effects of population ageing and medical resources can provide targeted guidance for policymaking, industrial development, and healthcare protection for healthy ageing in the future.

Earlier literature profiled the temporal and spatial trends of the ageing population and medical resources from temporal and spatial perspectives [1, 2]. There were, however, several limitations found in these studies, such as: a short period, limited spatial coverage, partial evaluation indicators, restricted prediction content, single prediction method, and limited prediction performance. In terms of duration, the time range of previous studies is relatively short. In terms of spatial coverage, prior studies have mainly been confined to a particular area [19, 20], requiring a more effective analysis of the distribution of medical resources at the national level and employing spatio-temporal statistics to investigate the spatio-temporal variation of medical resources. In relation to evaluation indicators, previous studies were restricted to single-level evaluation indicators such as the Gini Coefficient [19], Theil Index [21] and Atkinson Index [22], without considering practical guidance requirements. In terms of forecasting methods, historical studies have covered spatial aggregation [18], time series [1], kernel density estimation [23], and evolution forecasting [2], in addition to employing various time series forecasting methods [24, 25]. However, forecasting by a single method suffers from poor accuracy and lack of generalization.

Consequently, our work made refinements in the aspects of spatio-temporal distribution patterns, spatio-temporal interactions, the improved evaluation indicator, and a hybrid forecasting method to address current research problems. The first step was to include national data on the population ageing indicator and medical resource factors from 2011 to 2020. Furthermore, the methods of spatial autocorrelation [26, 27], Bayesian spatio-temporal model [18], and kernel density estimation [28] were applied to visualize evidence-based decision-making and identify spatial clustering characteristics of unbalanced medical resource allocation in an ageing population context. Additionally, the innovative indicator of ageing-resources (IAR) proposed in this paper was used to comprehensively measure the level of spatial allocation of population ageing and medical resources nationwide. Finally, a hybrid model (ETS-DNN) based on deep integrated time-series principles is also constructed in this paper to predict future trends in population ageing and medical resources. In sum, this paper is of great practical significance to provide reliable data support and scientifically feasible decision-making guidance for the goal of Healthy Ageing in China.

## Methods

**Fig. 1:**
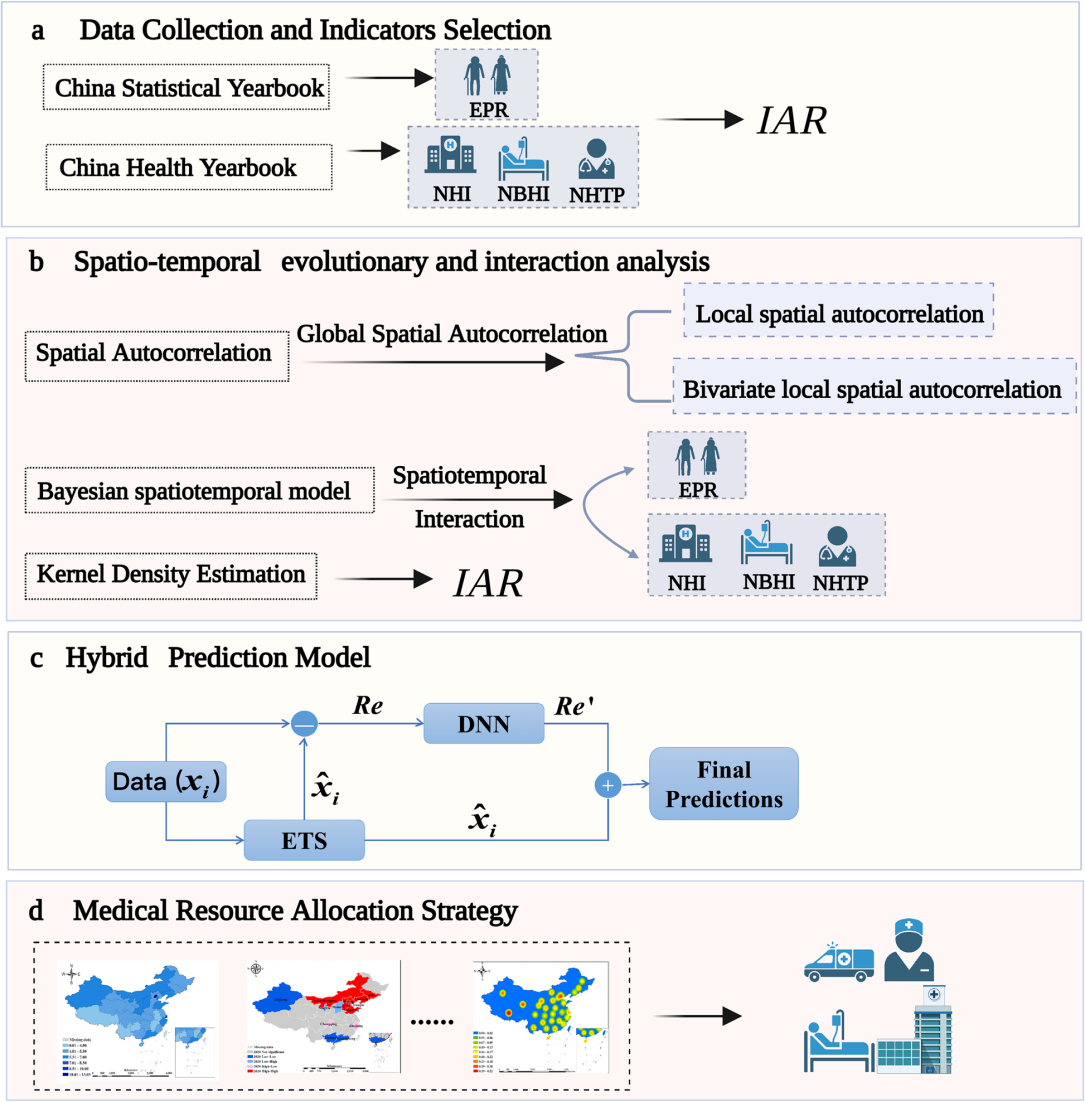
Main technical framework

Figure 1 illustrates the technical framework of this study. The methodology employed consists of the following stages: (1) data collection, (2) identification of spatio-temporal distribution patterns, (3) analysis of spatio-temporal interactions, (4) evaluation of the matching level of medical resources with the ageing population, and (5) simulation prediction. This study proposes recommendations for future medical resources allocation strategies based on the results of the preceding analysis.

### Data source and fundamental evaluation indicator selection

This study mainly examines the spatio-temporal evolution of population ageing and medical resources in mainland China. The fundamental evaluation indicators specifically include population ageing and medical resources, as shown in Table 1. In this context, population ageing is measured by the ratio of the elderly population aged 65 and over to the total resident population (EPR) [29]. The medical resource indicators are the number of health institutions per 1000 population (NHI), the number of beds in health institutions per 1000 population (NBHI), and the number of health technical personnel per 1000 population (NHTP). These three medical indicators measure healthcare, material, and human resources, respectively, and represent regional medical resource allocation measures. The higher the value of each indicator, the more abundant that medical resource is per capita. Due to the reliability and availability of a uniform data source, this study gathered data from China Statistical Yearbook (CSY) and China Health Statistical Yearbook (CHSY) for 31 regions in China (excluding Hong Kong, Macao, and Taiwan) from 2011 to 2020.

**Table 1:** Indicator selected in the study (2010–2020)

Indicator	Definition	Data source
EPR	The ratio of the elderly population (65 and over) to the total resident population	China Statistical Yearbook
NHI	The number of health institutions including hospitals, sanatoriums, health centers, etc	China Health Statistical Yearbook
NBHI	The number of beds in health institutions above	China Health Statistical Yearbook
NHTP	The number of health technicians per 1,000 population at the end of the year	China Health Statistical Yearbook

### Spatial autocorrelation analysis

This paper uses spatial autocorrelation analysis to research mainland China's agglomeration and spatial pattern of population ageing and medical resources. Specifically, spatial autocorrelation reflects the relevance between a regional unit's attribute value and a neighboring unit's same or different attribute value. It is a metric for evaluating the degree of aggregation in the spatial domain, and its measure is Moran's I. To quantify this aggregation property, global Moran's I, local Moran's I, and bivariate local Moran's I are introduced. GeoDa software (Version 1.18) is used for cluster analysis, and ArcGIS (Version 10.5) is used for mapping in this study.

Global spatial autocorrelation is a robust tool to capture the spatial clustering of variables. In this paper, we apply global Moran's I to measure the spatial autocorrelation of the population ageing indicator and medical indicator, which can be formulated as [30]:

1 $$I_{(global)} { = }\frac{{{\text{n}} \times \sum\nolimits_{i}^{n} {\sum\nolimits_{j}^{n} {W_{ij} } } \left( {x_{i} - \overline{x}} \right)\left( {x_{j} - \overline{x}} \right)}}{{\sum\nolimits_{i}^{n} {\sum\nolimits_{j}^{n} {W_{ij} } } \times \sum\nolimits_{i}^{n} {\left( {x_{i} - \overline{x}} \right)^{2} } }}$$

where $I_{(global)}$ represent the global Moran’s I statistic, *n* represents the number of regions in this paper, $x_{i}$ represents the value of the selected variable, $\overline{x}$ is the average value of all selected variables. The weight matrix $W_{ij}$ is described in an $\left( {n \times n} \right)$ matrix that illustrates the adjacency of spatial unit *i* to spatial unit *j*, where adjacency is assigned a value of 1 and non-adjacency is 0.

To identify the clustering characteristics of ageing and medical indicators by region, this paper uses local spatial autocorrelation based on positive global spatial autocorrelation [31]. Local Moran's I is calculated as follows [30]:

2 $$I_{(local)} = \frac{{\left( {x_{i} - \overline{x}} \right)}}{{S^{2} }}\sum\limits_{j} {W_{ij} \left( {x_{j} - \overline{x}} \right)}$$

where $I_{(local)}$ is the local Moran's I statistic and $S^{2}$ is the variance of local Moran's I.

Bivariate local spatial autocorrelation captures the spatial lagged correlation between the values of the ageing indicator *a* in *i*-th region and the neighboring values of medical resource indicators *b* (NHI, NBHI, and NHTP), which in turn reflects the spatial aggregation between the two. Bivariate local Moran's I is expressed as [30]:

3 $$I_{ab}^{i} = P_{a}^{i} \times \sum\limits_{j = 1}^{n} {W_{ij} \times p_{b}^{j} }$$

4 $$P_{a}^{i} = \frac{{X_{a}^{i} - \overline{X}_{a} }}{{\delta_{a} }}$$

5 $$P_{b}^{j} = \frac{{X_{b}^{j} - \overline{X}_{b} }}{{\delta_{b} }}$$

where $X_{a}^{i}$ is the value of indicator *a* located in spatial unit *i*, $X_{b}^{j}$ is the value of variable *b* located in spatial unit *j*. $\overline{X}_{a}$, $\overline{X}_{b}$ are the mean of the values taken for indicators *a* and *b*, $\delta_{a}$, $\delta_{b}$ are the standard deviations of the values taken by indicators *a* and *b*.

### Bayesian spatio-temporal model

With an understanding of the overall pattern, local trends, and lagged relationships between indicators of population ageing and medical resources in space, the Bayesian spatio-temporal model [24, 32] can analyze the interaction characteristics between variables from the perspective of spatio-temporal interactions [23, 33].

$\theta_{it}$ represents the relative risk ratio of the EPR for *i*-th region, i.e. the ratio of the actual EPR value for *i*-th region to the national baseline value in *t*-th year. Combining the three medical resource indicators, this can be expressed as:

6 $$\log \left( {\theta_{it} } \right) = \beta_{0} + \sum\limits_{k = 1}^{3} {\beta_{k} X_{kit} + \varphi_{i} + \Phi_{t} + \delta_{it} }$$

here, $\beta_{0}$ is the intercept; $\beta_{k}$ is the corresponding regression coefficient; $x_{kit}$ is the *k*-th medical resource indicator (*k* = 1, 2, 3)for *i*-th region in *t*-th year; $\varphi_{i}$ is the spatial effect, measured by the adjacency matrix W = ($W_{ij}$); $\Phi_{t}$ is the time effect; and $\delta_{it}$ is the interaction effect of time and space.

This paper performs statistical estimation of a Bayesian spatio-temporal effects model based on the Markov chain Monte Carlo method (MCMC) approach by using the CARBayesST package in R (Version 4.1.1). This model is shown to be convergent by its Geweke range of less than 1.96 [34] for all parameters.

### Improved the evaluation indicator

The application of fundamental evaluation indicators (EPR, NHI, NBHI, and NHTP) provides only a rudimentary assessment of the rate of population ageing and the per capita availability of medical resources. Present research [15, 19] on resource equity mostly uses the Gini coefficient and the Theil index. The Gini coefficient is used as a measure of general equity in resource allocation. However, it is incomplete and non-comparable. The Theil index can quantify the contribution of intra- and interregional variation to overall equity, but only measuring a single indicator or level may lead to biases. Thus, this paper constructs an innovative indicator of ageing-resources (IAR). The IAR is not a single-tier evaluation indicator, but a combination of three indicators of medical resources. With the IAR, the matching level can be measured in the spatial allocation of medical resources in different regions based on population ageing and holistic medical resources. The construction process is as follows.

Firstly, we construct relative spatial levels of ageing and medical resource indicators based on the spatial relative level parameters in the Bayesian spatiotemporal model. $\exp (S_{i}^{EPR} )$ and $\exp (S_{i}^{MR} )$ represent the ratio of the corresponding indicators in the *i*-th region relative to the national baseline level, which can be expressed as:

7 $$\exp (S_{i}^{EPR} ) = \frac{{\exp (\alpha^{EPR} + S_{i}^{EPR} )}}{{\exp (\alpha^{EPR} )}}$$

8 $$\exp (S_{i}^{MR} ) = \frac{{\exp (\alpha^{MR} + S_{i}^{MR} )}}{{\exp (\alpha^{MR} )}}$$

here, $S_{i}^{EPR}$ and $S_{i}^{MR}$ represent the spatial relative risk of EPR and the three medical resource indicators (NHI, NBHI, and NHTP) in that year, respectively. $\alpha^{EPR}$ and $\alpha^{MR}$ are constant parameters with non-informative priors, that is, the national baseline levels of the EPR and the three medical resource indicators.

Secondly, the spatial matching indicator $OR_{MR}$ of medical resources relative to EPR was constructed based on the concept of OR index in epidemiology.

9 $$\begin{gathered} OR_{MR} = \frac{{\exp (\alpha^{MR} + S_{i}^{MR} )/\exp (\alpha^{MR} )}}{{\exp (\alpha^{EPR} + S_{i}^{EPR} )/\exp (\alpha^{EPR} )}} \\ = \frac{{\exp (S_{i}^{MR} )}}{{\exp (S_{i}^{EPR} )}} \\ \end{gathered}$$

where, $OR_{MR}$ is the ratio of $\exp (S_{i}^{MR} )$ to $\exp (S_{i}^{EPR} )$ in relation to the national base level, which is conducive to expressing the adequacy of medical resource allocation levels based on an ageing population and reflecting a balanced allocation.

Thirdly, by introducing hyperparameters *p* and* q*, the three types of spatially matched indicators $OR_{MR}$ (NHI, NBHI, and NHTP) are integrated, and their weights are balanced as follows:

10 $$IAR_{(prior,i)} = (1 - p - q) * OR_{NHI} + p * OR_{NBHI} + q*OR_{NHTP}$$

Finally, considering the correlation of NHTP to EPR, after several pre-experimental parameter adjustments, the hyperparameters were set to $p = 1/4$, $q = 1/2$. Finally, the odds ratio of $IAR_{(prior,i)}$ to the provincial IAR mean value was processed:

11 $$IAR_{(st,i)} = \frac{{IAR_{(prior,i)} }}{{(\Sigma_{i}^{n = 31} IAR_{(prior,i)} )/n}}$$

Generally, if $IAR_{(st,i)} \ge 1$, the level of comprehensive medical resources in the *i*-th region is no less than its level of ageing (and vice versa), indicating a sufficient level of comprehensive medical allocation. Higher the value, the greater the sufficiency of the allocation of medical resources based on population ageing. Consequently, the spatial distribution of IAR was estimated as kernel density and mapped using ArcGIS (version 10.5) to explore the density of distribution of integrated healthcare resources in geographical space in response to ageing in each region.

### Hybrid ETS-DNN model

Currently, there are various time series forecasting methods based on linear and non-linear models or a combination of the two. Studies [35] have shown that combining linear and non-linear models can improve forecasting accuracy. This paper combines ExponenTial Smoothing (ETS) [36, 37] and Deep Neural Network (DNN) [38] to construct the hybrid prediction model ETS-DNN for forecasting indicators. In the proposed ETS-DNN model, the time series is assumed to be the sum of two variables, linear and non-linear. During the training process, we notice that using only ten years of data for the study was not sufficient for training the model. In this regard, we will use ETS to generate 'new samples' for supervised learning moving forward using a sliding time window approach. The ETS [39] considers the baseline and trend of the time series data and is suitable for trend prediction of the linear part. Besides, the DNN can reduce prediction errors by fitting and correcting the residual series that cannot be predicted by the linear part. The steps are as follows:

- (1) Actual values of each variable from 2011 to 2020 were applied to ETS, and forecasts were obtained for 2021–2030.
- (2) We add the trend information of the IAR to the actual and projected data from 2011 to 2020 and then exponentially smooth it to obtain a forecast value $\hat{x}_{i}$ that balances baseline and trend. $s_{i}$ expresses the weighted average of the current variable statistic $x_{i}$ and the smoothed value $s_{i - 1}$ containing the previous trend information $t_{i - 1}$, with a weighting factor of α. Equation (12) describes the process of trend smoothing, where the unsmoothed value of the trend is the current smoothed value $s_{i}$ minus the previous smoothed value $s_{i - 1}$. The trend is then smoothed exponentially by introducing the parameter β.
  12 $$s_{i} = \alpha \cdot x_{i} + \left( {1 - \alpha } \right) \cdot \left( {s_{i - 1} + t_{i - 1} } \right)$$
  13 $$t_{i} = \beta \cdot \left( {s_{i} - s_{i - 1} } \right) + \left( {1 - \beta } \right) \cdot t_{i - 1}$$
  14 $$\hat{x}_{i} = s_{i} + t_{i}$$
- (3) The DNN fits the residual error derived from the original time series data minus the predicted value of ETS $\hat{x}_{i}$.
- (4) Combining parts of ETS and DNN for integrated prediction. The evaluation metrics for the regression task include Mean-Square Error (MSE), Mean Absolute Error (MAE), Root-Mean-Square Error (RMSE), Mean Absolute Percentage Error (MAPE), and R-square (R^2^). The first four metrics represent the model fitting error, while R^2^ represents the model fitting trend. Generally, the smaller the error, the better the model fitting performance, and the larger the R^2^, the better the model's ability to predict actual data trends.

## Results

### Spatio-temporal distribution trends detection

**Fig. 2:**
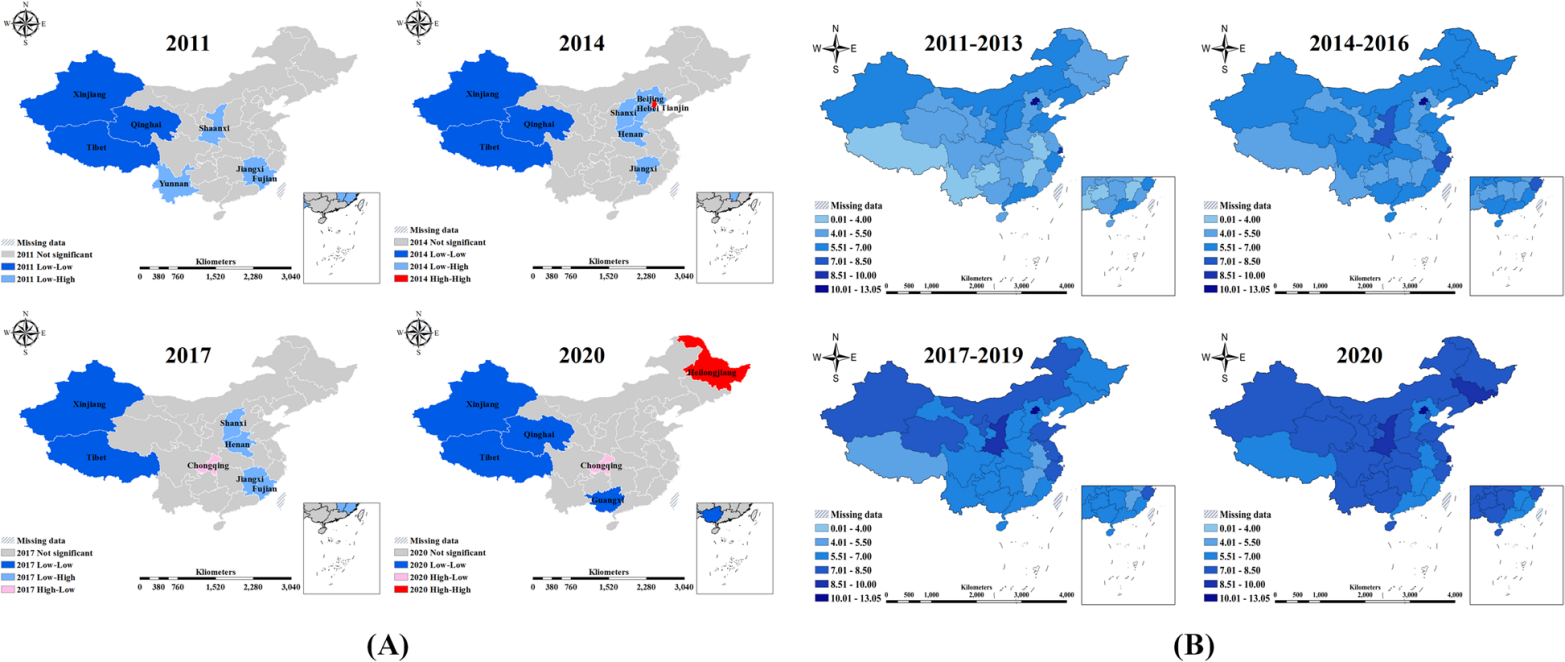
Spatio-temporal distribution trends of EPR and medical resources. Note: Plot (**A**) shows the EPR LISA plots for 2011, 2014, 2017 and 2020. Plot (**B**) presents NHTP as an example of spatial–temporal distribution trends in medical resource indicators

Population ageing is characterized by a general spatial agglomeration, with the global Moran's I value rising gradually from 0.16 (2011) to 0.43 (2020). The local spatial characteristics are dominated by high-value agglomeration and low-value agglomeration patterns, with low-value agglomerations mostly in Western China and high-value agglomerations mostly in Eastern China, as detailed in Fig. 2A. As a result, Eastern China shows a high level of ageing, and Western China has a low level of ageing.

China's medical resources show a slow year-on-year increase, with imbalances in the supply of medical resources across regions. Western China shows relatively high levels of NHI and NBHI, while Eastern China has high relative levels of NHTP. In 2020, the regions with the highest NHTP levels included Beijing (12.61), Shaanxi (9.2), and Jilin (8.81), while the regions with the lowest NHTP levels include Tibet (6.23), Jiangxi (6.33) and Guangdong (6.58), as detailed in Fig. 2B.

### Spatio-temporal interaction analysis

An analysis of the correlation between ageing and health care resources shows that: an increase in EPR corresponds to a risk of NHTP of 1.041 (95% CI: 1.020,1.067), while an increase in EPR corresponds to a risk of NHI of 0.983 (95% CI: 0.950,1.002) and an increase in EPR corresponds to a risk of NBHI of 1.008 (95% CI: 0.998,1.022). Thus, there is a definite positive spatio-temporal interaction between EPR and NHTP, i.e., each 1% increase in EPR is associated with a 1.041% increase in NHTP to meet response needs.

**Fig. 3:**
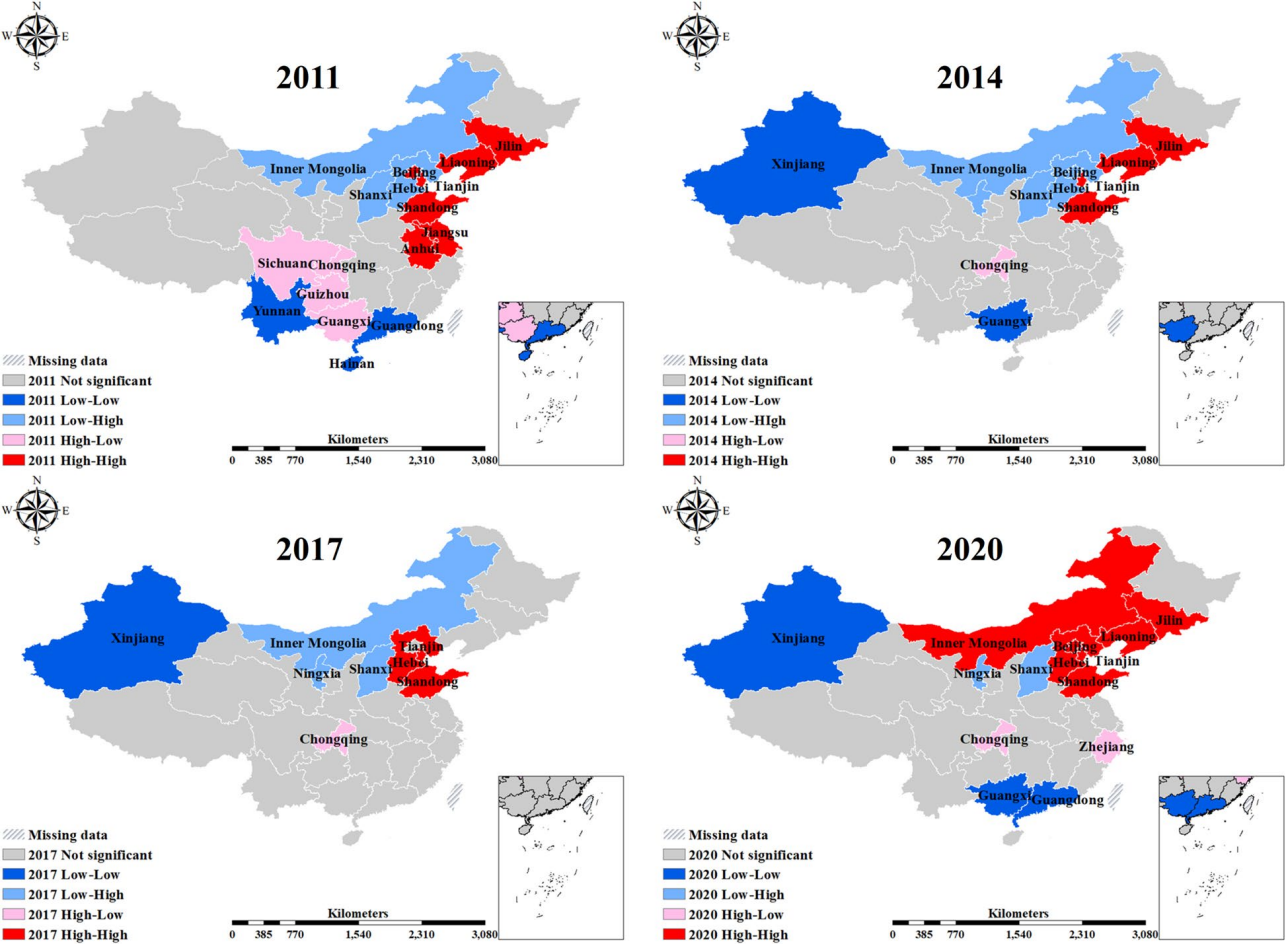
Spatial distribution and variation of BiLISA between EPR and NHTP. Note: The paper shows the BiLISA plots between EPR and NHTP for 2011, 2014, 2017 and 2020

A bivariate local spatial autocorrelation analysis of the interaction effects between ageing and medical resources is performed in this paper. The medical resource indicator in the paper takes NHTP as an example, as shown in Fig. 3. The results show a significant H–H and L-L positive correlation between EPR and lagged NHTP. NHTP and EPR are high in East China, and low in West China, while BiLISA indicates a spatial movement toward the north.

### Improved indicator IAR trend analysis

**Fig. 4:**
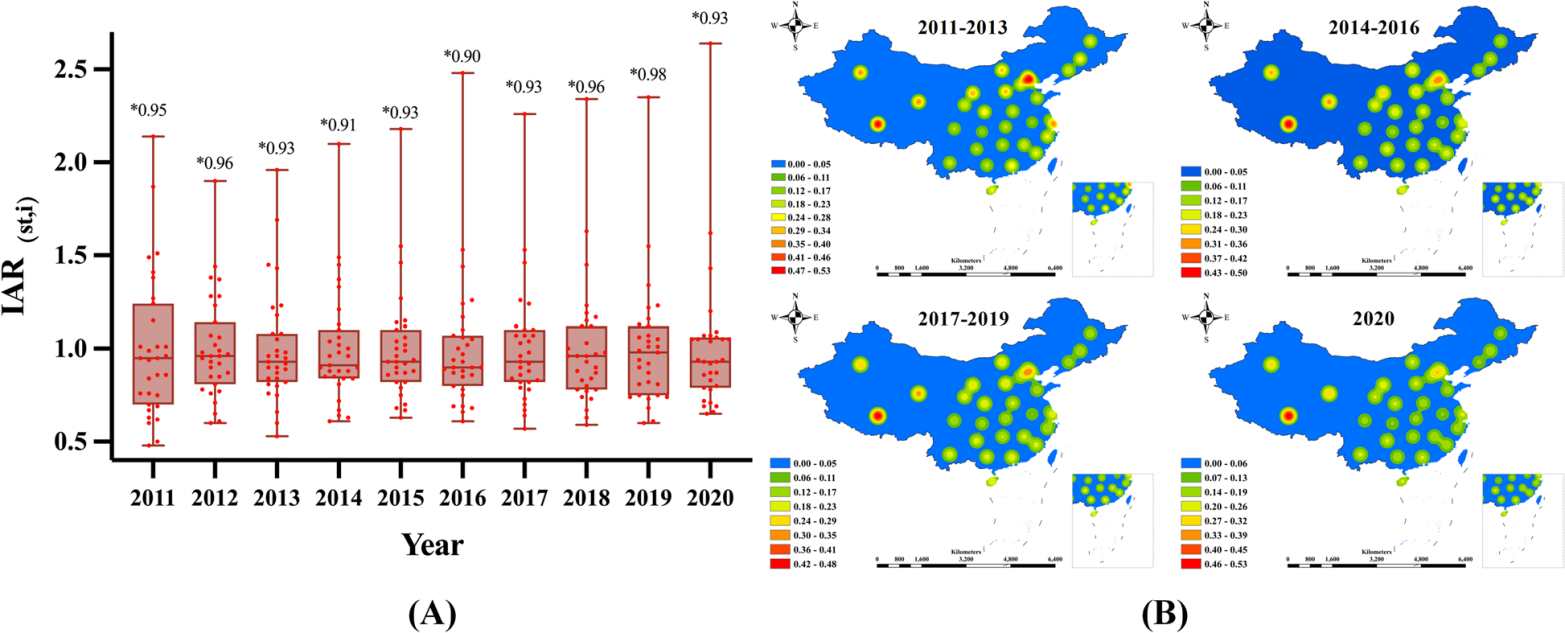
IAR Distribution by Region in China from 2011 to 2020. Note: Plot (**A**) shows the Annual IAR Box Chart by Province in China; * refers to the median IAR value of each year. Plot (**B**) presents the kernel density distribution of IAR by region; the maps reflect the hotspot areas of the IAR

In China, there are only 9–12 provinces with comprehensive medical allocation sufficiency based on population ageing (i.e., $IAR_{(st,i)} \ge 1$), from 2011 to 2020. This indicates that most regions are lacking comprehensive medical resources to cope with the present. Provinces with sufficient IAR are concentrated in North China (Beijing, Shanxi, Inner Mongolia), Southwest China (Tibet, Guizhou, Yunnan), Northwest China (Shaanxi, Qinghai, Ningxia, Gansu), and South China (Guangdong, Hainan.) In 2020, the median IAR for the 31 regions in China is 0.93, as shown in Fig. 4A.

Based on the improved evaluation indicator, this paper estimated the kernel density of the spatial distribution of IAR, as shown in Fig. 4B. The results show that the IAR levels are relatively high in Western China (Xinjiang, Tibet, Qinghai), North China (Beijing, Tianjin) and Yangtze River delta (Shanghai, Zhejiang). In terms of time trends, the IAR levels in North China and the Yangtze River Delta region weakened over time, with a more pronounced downward trend in the Yangtze River Delta region.

### Simulated forecast

The IAR data from 2011 to 2018 is the training set, and the IAR data from 2019 to 2020 is the test set. This paper compares the prediction effects of various regression models. The results show that the ETS-DNN based on the concept of deep integration has excellent prediction performance (main evaluation metrics), as shown in Table 2.

**Table 2:** Evaluation of regression prediction model results

Methods	Algorithm	MSE	MAE	RMSE	MAPE	R^2^
Time-series regression method	ETS	0.0044	0.0473	0.0664	4.6515	0.9634
Machine learning model	LR	0.0115	0.0754	0.1073	8.0892	0.9044
	DTR	0.0042	0.0455	0.0648	4.4959	0.9651
	SVR	0.0138	0.0893	0.1177	9.2090	0.8850
	KNR	0.0052	0.0500	0.0722	4.8603	0.9567
	ETR	0.0042	0.0455	0.0648	4.4959	0.9651
Integrated learning model	RFR	0.0039	0.0431	0.0621	4.1990	0.9679
	ADBR	0.0042	0.0456	0.0649	4.5277	0.9651
	GBR	0.0042	0.0455	0.0648	4.4949	0.9651
	BR	0.0039	0.0444	0.0624	4.3633	0.9677
	XGB	0.0042	0.0454	0.0647	4.4900	0.9652
Deep learning model	MLPR	0.0228	0.1105	0.1509	12.3355	0.8108
	DNN	0.0038	**0.0422**	0.0613	**4.0099**	0.9688
Deep integration of time-series model	ETS-DNN	**0.0034**	0.0454	**0.0582**	4.6398	**0.9719**

*ETS* ExponenTial Smoothing Method, *LR* Linear Regression model, *DTR* Decision Tree Regressor, *SVR* Support Vector Regression, *KNR* K-Nearst Neighbors Regressor, *ETR* Extra Tree Regressor, *RFR* Random Forest Regressor, *ADBR* AdaBoost Regressor, *GBR* Gradient Boosting Regressor, *BR* Bagging Regressor, *XGB* XGBoost, *MLPR* Multi-layer Perceptron Regressor, *DNN* Deep Neural Networks

**Fig. 5:**
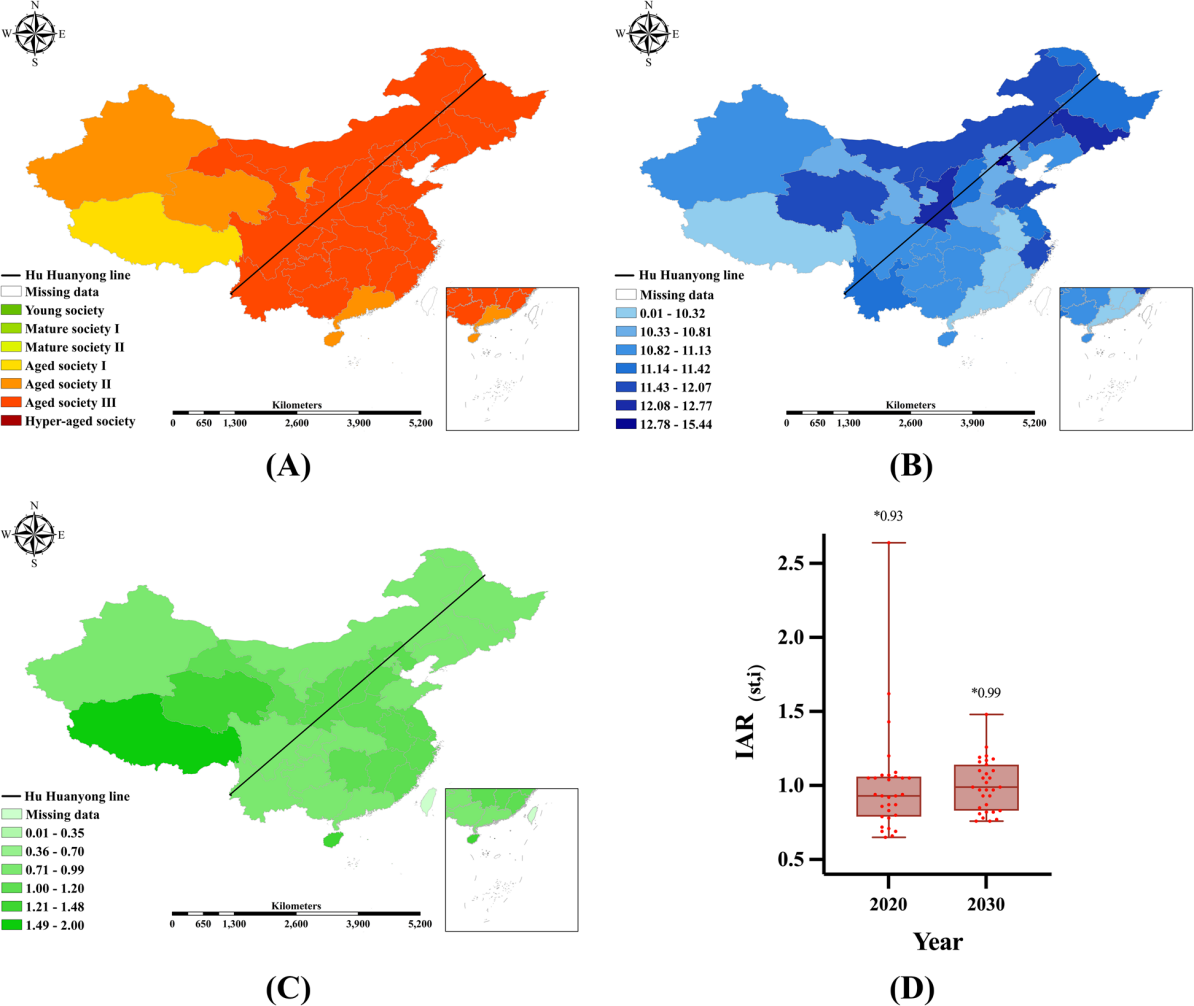
Distribution of projected EPR, NHTP, and IAR in 2030. Note: Projections of EPR, NHTP and IAR levels in 2030 with ETS-DNN. **A** EPR; **B** NHTP; **C** IAR; **D** Distribution of IAR by Province (2020 Vs 2030), * refers to the median IAR value of each year

Based on the ETS-DNN method, the distribution of EPR, NHTP, and IAR from 2021 to 2030 is predicted, as shown in Fig. 5, and a brief analysis of the three trends is provided. (1) Regarding EPR trends, Chongqing was China's most severely ageing province from 2011 to 2017; since 2020, Liaoning has topped the list. In 2030, except for Tibet, mainland China enters Aged Society II [10.00,14.00) across the board; 21 provinces enter Aged Society III [14.00,20.00); four provinces—Liaoning, Shanghai, Chongqing, and Sichuan—enter Hyper-aged society, with Liaoning having the highest EPR (20.99%). (2) NHTP trends show a faster rate of increase. In 2030, spatially high and low-value provinces will remain consistent with 2020. China's highest NHTP levels are in Beijing (15.44), Shaanxi (12.77), and Jilin (12.42), while its lowest levels are in Tibet (9.80), Jiangxi (9.90), and Guangdong (10.17). (3) In terms of IAR trends, the median IAR in 31 provinces increased from 0.93(2020) to 0.99(2030). It is pertinent to note that although the median IAR values has not yet reached 1, the overall trend is positive—the number of provinces with $IAR_{(st,i)} \ge 1$ has increased to 15.

## Discussion

With an increasingly ageing population, the demand for medical resources in China is steadily growing. The ability of medical resources to adapt to the growth rate of ageing has become an essential issue for most developing countries. A global study of population ageing [2] demonstrates that ageing is a worldwide issue, but the characteristics of population ageing vary from region to region. China is the fastest ageing country, indicating challenges for public health policy. Therefore, it is relevant to examine the patterns of medical resource allocation in China in the context of population ageing.

The distribution patterns and variations in the elderly population and medical resources have different characteristics in spatio-temporal distribution trends detection. Eastern China is highly ageing and generally abundant in medical resources. Western China is mildly ageing and generally limited in medical resources but outperforms Eastern China when it comes to per capita allocation of medical resources. NHTP shows a more pronounced upward trend, representing the most critical element of healthcare resources. A city-based study [19] shows that the overall equity in the allocation of medical resources is equitable, but there are significant regional differences in their distribution. According to this study, population ageing shows clustering characteristics by province, with high-value and low-value clustering patterns dominating in general. A longitudinal study of medical resources during a decade of China's new healthcare reform [40] provides insight into the spatial autocorrelation of medical resources. It finds that medical resources are unevenly distributed in China. This is consistent with our study's findings. Xinjiang, Qinghai, and Tibet are spatially linked in a patchy distribution, implying that the Western provinces show aggregation characteristics with low levels of population ageing. Tianjin and Heilongjiang are surrounded by neighboring regions with high levels of population ageing, while the high-value agglomeration area changes from Tianjin (2014) to Heilongjiang (2020). These findings have important implications for the development of public health policies and the allocation of medical resources.

To our knowledge, this study is the first to assess the spatio-temporal interaction between EPR and medical resources at the provincial levels based on a Bayesian spatio-temporal model. Earlier literature [18], using the Bayesian geo-detector model, showed that from 2008 to 2017, the spatiotemporal matching degree between the EPR and medical personnel and facilities (beds in hospitals) in mainland China was generally quite low. Whereas our results further reveal a relatively solid spatio-temporal interaction between EPR and NHTP. To explore their spatial aggregation further, this study conducted a bivariate spatial autocorrelation analysis of EPR and NHTP. The study finds that Eastern China enjoys a higher degree of EPR and NHTP while Western China obtains a lower degree of EPR and NHTP. More specifically, Inner Mongolia has changed from a long-term negative L–H correlation to a positive H–H correlation in 2020, suggesting a deepening population ageing in Inner Mongolia and a shift to a positive correlation with the number of health technicians per capita. Jiangsu, Shandong, Beijing, and Jilin show a robust positive correlation between EPR and NHTP. This suggests that these regions also have a relatively high number of health technicians per capita despite their high ageing level. Allocating medical technicians in conjunction with population ageing characteristics is key to scientific deployment decision-making. A significant measure to deal with population ageing is the Talent Introduction Policy for provincial areas with high EPR. Additionally, it reduces the burden on pensions and the pressure on the public finances as a result of population ageing. To meet the demand for medical personnel, the government should implement the Talent Introduction Policy, and universities should increase enrolment in healthcare and nursing professions.

The main indicators currently used to study resource equity are the Gini coefficient and the Theil index, but they have some limitations [15, 19]. The Gini coefficient is not complete and comparable, while the Theil index only measures a single indicator. In this paper, we propose an innovative indicator of ageing-resources (IAR) that considers the impact of both medical resources and population ageing in addressing these shortcomings. It can provide a more comprehensive and accurate assessment of the matching level between medical resources and the ageing population, providing a more holistic evaluation. The IAR indicator proposed in this paper is beneficial in measuring the ability of a certain region's medical resources to respond to ageing. The IAR in Eastern China is generally inferior to that in Western China. The hotspot regions of the IAR are identified based on the kernel density analysis, revealing higher matching levels in Northwest and North China and the Yangtze River Delta. The higher IAR in Northwest China is attributed to a sparser population density and a lower relative level of population ageing, although the total amount of medical resources is not dominant. North China and the Yangtze River Delta have relatively high IAR, but there is a year-on-year downward trend owing to the faster increase in population and the rising relative level of population ageing in these economically developed regions. Overall, the large population density and high demand for medical resources in the East provide support for future public health interventions. The median IAR value of the 31 provincial regions is predicted to increase from 0.93 (2020) to 0.99 (2030). Although the median IAR value has yet to reach 1, the overall level of integrated medical resource allocation for an ageing population is increasing. To improve the comprehensive medical resource allocation based on population ageing, China should consider regional differences and local variation trends. It is the government’s responsibility to provide policy support for the construction of an ageing protection system and for the development of health industries. For instance, it is necessary to allocate more medical resources to Eastern regions with a lower IAR and to strategically respond to the Western regions with a higher IAR for a future super-aged society [15]. Provinces with abundant medical resources can offer assistance to neighboring disadvantaged provinces [41].

The simulation forecast analysis indicates that the national population ageing continues to deepen, potentially triggering a more significant social burden. In previous studies [16, 19, 41], equity in medical resource distribution has typically been examined through descriptive or evaluative methods, while lacking predictive methods for anticipating future developments. In contrast, this paper proposes a hybrid forecasting model, ETS-DNN, which considers the baseline and trend of time series data and utilizes linear and non-linear fusion approaches. ETS-DNN can effectively predict the trend of ageing and medical resources and provide a rationale for medical resource allocation decisions. Additionally, we analyzed data from the period of 2011–2020, which is more recent than the data used in previous studies [21, 41], providing a more up-to-date understanding of the issue at hand. With this approach, we can better predict future medical demands and allocations for the elderly, and provide solid reference for relevant organizations. The forecasting results show that the provinces with more sufficient levels of comprehensive medical allocation for population ageing have changed spatially, with the new regions of Eastern China (Shanghai, Jiangsu, Zhejiang, Anhui, and Jiangxi) and Central China (Hunan and Henan) being added from Northern, Southwest and Northwest China. In light of rapidly increasing ageing, the growth in medical resources in Eastern regions has been significantly slower than the ageing upward trend, revealing the necessity for intervention. A study [42] revealed that case fatality rates showed an age-related gradient (60–69:1.93%; 70–79:4.28%; ≥ 80: 7.8%). Therefore, to meet the routine access needs of the elderly, it is essential to strengthen macro control of the allocation of medical resources.

Our study also has several limitations. Firstly, the data incorporated in this paper are sampling data with limited application, and more comprehensive census data may be considered in the future. Further, this paper focuses on provincial administrative regions, and a finer spatial scale may be considered in the future. Finally, only ten years of data are included in this paper, and more years will be incorporated to enhance forecasting accuracy.

## Conclusion

In conclusion, this study provides a comprehensive examination of the interplay between population ageing and medical resources, and highlights the presence of a spatio-temporal interaction effect between them. The paper also introduces a novel evaluation indicator that reflects the matching level of medical resources to population ageing, underscoring the significance of addressing the challenges posed by an ageing society and the importance of nurturing a skilled health workforce. Utilizing an ETS-DNN approach, the study forecasts the future trends of ageing, medical resources and the IAR, offering valuable insights for potential policy interventions. The findings indicate that eastern China has a higher concentration of both medical resources and an ageing population compared to the western region. It is thus imperative to formulate a regionalized ageing security system and health service industry that caters to the demands of a future hyper-aged society.

## Data Availability

The datasets that support the findings of this study are available in the corresponding year’s China Statistical Yearbook (CSY) and China Health Statistical Yearbook (CHSY). The former can be hyperlinked to “http://www.stats.gov.cn/tjsj/ndsj/”, and the latter can be accessed at “http://www.nhc.gov.cn/mohwsbwstjxxzx/tjzxtjsj/tjsj_list.shtml”. Given that the corresponding yearbooks for this study are published for different years, direct hyperlinks may not be available and may change annually. Hence, please feel free to contact corresponding authors for data requests.

## Abbreviations

CSY: China Statistical Yearbook
CHSY: China Health Statistical Yearbook
EPR: Elderly population ratio
NHI: The medical resource indicators are the number of health institutions per 1000 population
NBHI: The number of beds in health institutions per 1000 population
NHTP: The number of health technical personnel per 1000 population
IAR: Indicator of ageing-resources
OR: Odds ratio
ETS: ExponenTial Smoothing
DNN: Deep Neural Network
